# Context-dependent influence of path integration on nest plume following in desert ants

**DOI:** 10.1016/j.isci.2026.116523

**Published:** 2026-06-25

**Authors:** Richard Spehr, Johanna Eschenhagen, Markus Knaden

**Affiliations:** 1 Department of Evolutionary Neuroethology, Max Planck Institute for Chemical Ecology, Hans Knoell Strasse 8, Jena 07745, Germany

**Keywords:** Entomology, Biophysics, Complex systems

## Abstract

The desert ant *Cataglyphis fortis* forages individually for dead arthropods in the Tunisian saltpans. Homing ants return to their nest by following a path integration (PI) derived home vector and finally pinpoint the nest entrance by tracking the nest’s carbon dioxide plume. However, as long as PI tells the homing ant that it has not reached its nest’s vicinity yet, plume following is inhibited. This avoids an ant entering foreign nests en route. If homing ants cannot find their nest, they begin a systematic search that results in the formation of a search-derived PI vector pointing toward the nest’s location. Here, we show that foraging-derived but not search-derived PI vectors inhibit plume following. Interestingly, although searching ants experience increased heat stress, heat stress alone cannot explain the observed effects. We conclude that PI vectors can inhibit plume following in homing but not in nest-searching ants.

## Highlights

• Whether or not to follow nest plumes in *Cataglyphis fortis* is context dependent
• Homing foragers that are still far from home do not follow nest plumes
• Ants that arrive home and start a nest search always follow nest plumes
• Heat stress alone does not induce nest-plume following in ants

## Graphical abstract

**Figure:**
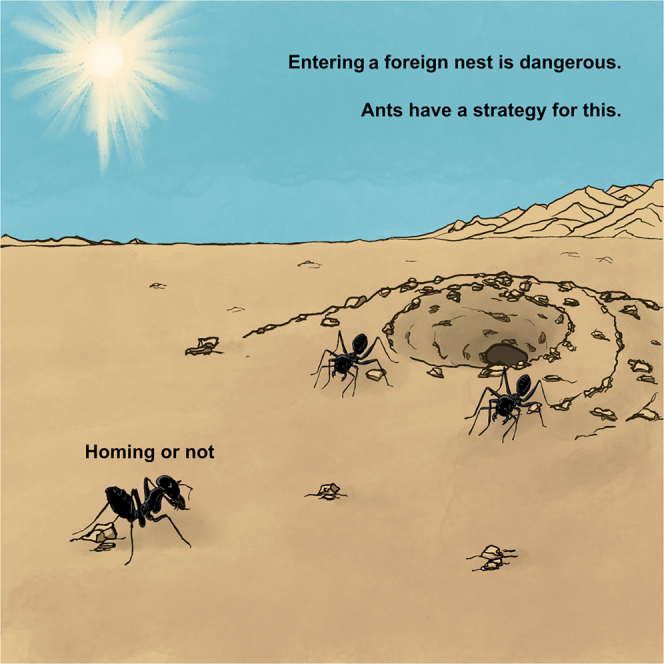

## Introduction

A wide variety of navigation skills can be found across the animal kingdom, which are often crucial for survival. These range from long-distance navigation using the Earth’s magnetic field^1^^,^^2^^,^^3^^,^^4^ and a time-compensated sun compass,^5^ as in the case of migratory birds or moths and butterflies, to short-distance navigation using pheromone trails, as in the case of many ants.^6^^,^^7^ A striking example of navigation skill is the desert ant *Cataglyphis fortis* that inhabits the flat Tunisian salt pans, where it individually forages for dead arthropods. The even distribution of food and the intense heat make trail pheromones unsuitable for this species.^8^ Instead, *C. fortis* relies on path integration (PI) during its foraging journeys,^9^ which often cover several hundred meters.^10^ By using a sky compass to determine their walking direction^9^ and a step integrator to calculate their distances covered,^11^ PI provides ants with a continuously updated vector pointing back to their nest.^9^ However, PI is error-prone, and despite guiding homing ants to the vicinity of their nest, it does not necessarily guide them directly to the often inconspicuous nest entrance. Therefore, in addition to PI, homing ants use visual cues to locate their nest^8^ and finally enter it by following a carbon dioxide plume emanating from the nest entrance.^12^ As aggression between different colonies of this species is high,^13^ ants must avoid following carbon dioxide plumes from neighboring nests that they may encounter during their homing runs. To prevent this, homing ants ignore nest plumes for as long as their PI vector indicates that they are still far from home.^12^

When the ants fail to find their nest using PI, they switch to a systematic search pattern consisting of a series of ever-increasing, centered loops.^14^ These loops are generated by an underlying spiral search program that is periodically interrupted by returns to the starting point of the search, expanding the overall area covered over time. In order to keep track of their position relative to the starting point of the search, the systematic search is governed by PI, resulting in the formation of a search-derived PI vector pointing at the PI-defined nest position.^14^

During a nest search, ants should always be prepared to follow their nest’s plume, regardless of their distance from the PI-defined nest position. Therefore, we hypothesize that a search-derived home vector should not inhibit plume following in ants, even when it is long.

## Results

### Foraging-derived path integration controls plume following in homing ants

**Figure 1:**
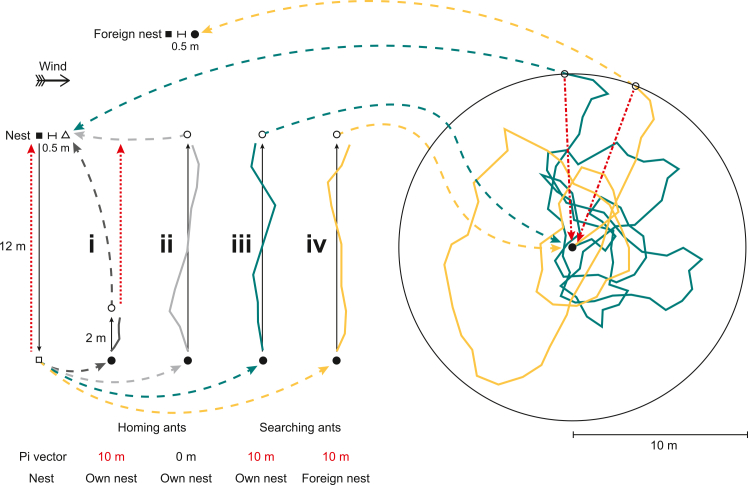
Experimental paradigm (i–ii) Homing ants; ants from a focus nest (black square) are trained to a feeder (open square), from where they are displaced (dashed lines) to a remote field (distance between feeder and release point (black circle), ca 50 m). Ants are allowed to run off their home vector either by 2 m (i, solid dark gray trace), or completely (ii, solid light gray trace) before they become recaptured (open circle) and released 50 cm downwind of their own nest (open triangle). Red dotted lines represent foraging-derived PI vectors. (iii–iv) Searching ants; ants from a focus nest (black square) are trained to a feeder (open square), from where they are displaced to a remote field. Ants are allowed to run off their home vector completely before they become recaptured (open circle) and released again in the absence of any nest. As soon as the ants reach 10 m distance from the release point, they become recaptured again and are released 50 cm downwind of either their own (iii, green trace) or of a foreign (iv, yellow trace) nest. Red dash-dotted lines represent search-derived PI vectors.

We first tested whether homing ants would respond to and follow their own nest’s plume when they cross this plume despite still having a PI vector of 10 m length, derived from the foraging run. To do so, ants were captured at a feeder (nest-to-feeder distance, 12 m) and released to a remote field where they were allowed to partially run off their PI vector by 2 m. The ants were recaptured and then, with the remaining PI vector of 10 m, released into the nest plume 0.5 m downwind of their nest (Figure 1_i_).

**Figure 2:**
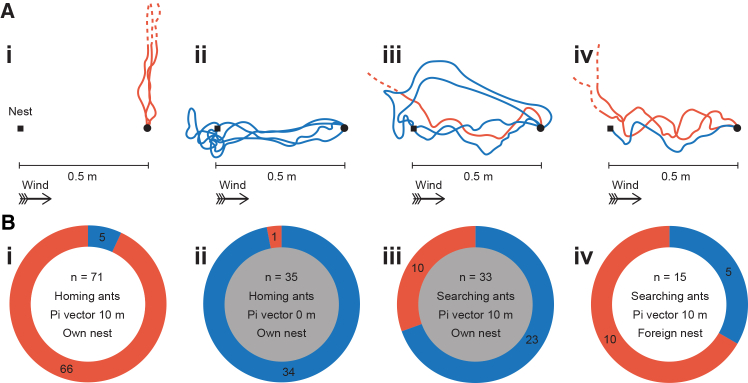
Nest plume following in homing and searching ants (A) Example traces of ants that were released 0.5 m downwind of their nest entrance. (B) Distribution of ants that entered (blue) or did not enter (red) the nest entrance. Blue traces, ants entered the nest; red traces, ants did not enter the nest. i (ii); homing ants with a path integration vector of 10 m (0 m) length; iii (iv) searching ants with a path integration vector of 10 m length tested at their own (a foreign) nest. Ants in experiments ii–iv (i.e., with run-off vector or with a search-derived vector of 10 m) enter the nest significantly more often than ants in experiment i (i.e., with a foraging-derived vector of 10 m; Fisher’s exact test with Bonferroni-Holm correction for repeated comparisons, for all individual *p* values and the corresponding corrected levels of significance (α) as well as odds ratios and confidence intervals see Table S1). The significant differences with respect to experiment i are shown in gray. For raw data, see Data S1.

Over 90% of all tested ants ignored the nest plume and instead followed their PI vector on a rather straight line, guiding them away from their nest (Figures 2A_i_ and 2B_i_).

When displaced ants were first allowed to run off their PI vector completely before being recaptured and released into the plume (Figure 1_ii_), 97% followed the plume immediately (Figures 2A_ii_ and 2B_ii_). We conclude that in line with,^12^ the length of the foraging-derived PI vector dictates whether a homing ant follows the plume of its nest.

### Search-derived PI vectors do not control plume following

When a homing ant, after having run off its PI vector, does not find its nest immediately, it engages in a systematic search that maximizes its chance to finally locate its nest entrance.^14^^,^^15^ While doing so, the ant starts building up a new PI vector, the search-derived PI vector. This vector is continuously updated and is pointing toward the location where the ant expects the nest to be.^14^ It ensures that the search remains centered on this location, i.e., that the ant during its search does not lose track of where the nest was originally indicated by PI.

Having reconfirmed that the foraging-derived PI vector controls nest-plume following, we hypothesized that a search-derived PI vector would not do so, as an ant would otherwise be unable to enter its nest during its nest search. We therefore again let ants run off their home vectors completely but then let them start their systematic nest search. After they had exhibited this search for a while and had established a new search-derived PI vector of 10 m in length (Figure 1_iii_), the ants were displaced downwind of the nest entrance, and their reaction to the nest plume was tested. Despite having a PI vector length of 10 m, 70% of these ants now ignored their PI information and followed the plume into the nest (Figure 2B_iii_). This leads us to assume that the assessment of foraging-derived and search-derived PI vectors differs.

Nest plumes in *C. fortis* are known to contain carbon dioxide, which acts as an attractant during plume following.^12^ As all *C. fortis* nests emit carbon dioxide, all nest plumes are potentially attractive to homing ants, and the ants’ foraging-derived path integrator prevents them from entering wrong nests. Therefore, in homing ants, PI is not only used for navigation, but is also considered to be involved in security.^12^ In conclusion, we asked whether ants that had built up a PI vector of 10 m during their search would also enter a foreign nest (Figure 1_iv_). We decided on a foreign nest that, such as the focus nest, was situated in the featureless center of the salt pan and flattened its nest hill to remove any obvious visual cues that would differentiate it from the focus nest. We then let homing ants from the focus nest run off their PI vector in a remote field, let them search for a while, and again captured them when their search-derived PI vector reached a length of 10 m. When we released these ants 0.5 m downwind of the foreign nest, most of the ants first followed the plume and pinpointed the nest entrance. At some point, the majority of ants turned away from the nest. However, one-third of the ants followed the plume and even entered the foreign nest (Figure 2B_iv_), where they were immediately attacked by resident ants, probably resulting in their death.

### Time differences do not account for differences in plume-following behavior

Next, we asked whether the origin of the PI vectors (i.e., whether they are derived from foraging or nest searching) really governed their different effects on plume following. As the procedure to build up a search-derived vector took longer than the procedure for the home-derived vector in our experiments, the different amounts of time or heat stress to which the ants were exposed could also have been driving factors.

**Figure 3:**
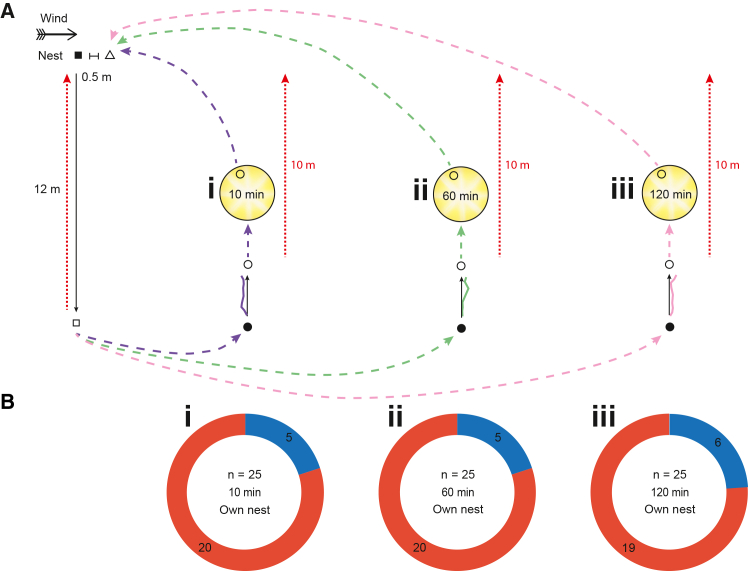
Effect of sun exposure (A) Experimental paradigm. (i-v) Homing ants; ants from a focus nest (black square) are trained to a feeder (open square), from where they are displaced (dashed lines) to a remote field (distance between feeder and release point (black circle), ca 50 m). Ants are allowed to run off their home vector by 2 m. After having run off the 2 m of their PI vector, ants were kept in a sun-exposed arena for 10 min (i), 60 min (ii), or 120 min (iii) before they were recaptured and displaced 50 cm downwind of their nest (open triangle). (B) Distribution of ants that entered (blue) or did not enter (red) the nest entrance after being exposed to 10 (i), 60 (ii), or 120 (iii) heat stress. Red dotted lines represent the foraging-derived PI vectors. For statistical analysis, a Fisher’s exact test with Bonferroni-Holm correction for repeated comparisons with the control data (shown in Figure 2B_i_) was performed. None of the groups differed significantly from the control group (shown in Figure 2Bi and Table S1). For raw data, see Data S1.

To test for the influence of these potential factors on plume-following behavior, we captured ants at the feeder, let them run off 2 m of their PI vector, and let them wait in arenas for 10 min (Figure 3A_i_, i.e., the time that it usually took for ants to build up a search vector of 10 m length). Afterward we released the ants into the nest plume 0.5 m downwind of their nest.

When doing so, 80% of the tested ants ignored the nest plume (Figure 3B_i_). Apparently, the 10 min that it took the ants to build up their search-derived PI in Figure 1_iii_ did not induce their willingness to follow the nest plume.

### Heat stress does not account for differences in plume-following behavior

We wondered whether being exposed to extreme heat stress might motivate an ant to follow a nest plume. To test the potential effect of heat stress, we exposed homing ants for even longer times to the sun. We again captured ants at the feeder, let them run off 2 m of their PI vector and let them wait in arenas, this time for 60 min (Figure 3A_ii_). Although the genus *Cataglyphis* has been shown to have adaptations to extreme heat, such as alterations in their heat shock protein response,^16^ the death of twelve ants during this procedure underscored the stress to which the animals were exposed here. The surviving ants were released into the nest plume 0.5 m downwind of their nest. Interestingly, again 80% of the ants ignored their nest plume and decided against the safety of the nest Figure 3B_ii_) and rather continued in the direction of their remaining PI vector (data not shown). Even after being exposed to heat stress for 120 min (Figure 3A_iii_, fourteen ants did not survive the procedure), 76% ignored the plume of the safe nest (Figure 3B_iii_). The percentage of ants that entered the nest did not vary from ants that were not exposed to additional heat stress at all (Figure 2B_i_). It seems that the length of time that homing ants were exposed to heat stress did not affect their willingness to follow the nest plume.

## Discussion

Therefore, we conclude that it is mainly the origin of the PI vector that dictates whether an ant follows a nest plume. When returning from long foraging trips, a foraging-derived home vector inhibits plume following, preventing the ants from entering foreign nests. Even ants that are exposed to extreme heat stress on their foraging journey will not enter any nest they encounter, as long as they have a remaining long foraging-derived PI vector. Apparently, they would rather risk dying from heat than from being killed in a foreign nest. Only after arriving at the supposed position of their nest and starting their systematic nest search will the ants ignore their (now search-derived) PI vector and follow whatever nest plume they encounter. Other animals such as the desert isopod *Hemilepistus reaumuri,* face similar environmental or predatorial risks.^17^ During its foraging runs, it avoids the burrow of its predator, the scorpion *Scorpio palmatus,* and adjusts this avoidance based on the number of scorpion-derived cues it detects.^17^^,^^18^ Similar to the ants, the fiddler crab *Uca pugilator* also finds its way back to the burrow by PI.^19^ Foraging fiddler crabs sometimes need to decide whether they continue feeding or return to their burrow to defend it against an approaching conspecific. Like in the ants, this decision is partly governed by PI. By comparing their own distance (based on PI) with the distance between the intruder and the burrow (based on vision) the crab adjusts its behavior in relation to the perceived risk, initiating return and defense when necessary.^20^ Our findings suggest that in *C. fortis* plume, following is not governed by a perceived risk—as otherwise ants exposed to increased heat stress should have followed any nest plume. Instead, plume following seems to be governed by the length of the PI vector and the context. Ants that return from a foraging trip do not follow nest plumes until they have run off their home-directed PI vector. This prevents them from ending up and being killed in foreign nests along their way home. However, if they do not find their nest immediately after they have run off their PI vector, they start a systematic search, during which they build up again a PI vector pointing at the starting point of the search. This vector, regardless of its length, does not prevent plume following, as during its nest search, an ant should always be ready to pinpoint its nest when detecting the nest’s plume. Whether the foraging-derived vector and the search-derived vector are two independent vectors, of which the former inhibits plume following, and the latter does not, remains unclear. Alternatively, it could be a single PI vector that becomes run down to zero during homing and builds up again during nest search. In this case, it would just be the context of either homing or searching which finally dictates whether an ant with a long home vector follows a nest plume. In the Australian desert ant, *Melophorus bagoti*, PI has been shown to be involved in disposing of garbage outside its nest entrance.^21^ Therefore, it would be interesting to investigate whether and how PI in contexts other than foraging and nest searching interferes with plume following in ants. Furthermore, homing ants, especially during the last meters before they enter their nest, often meet and antennate with nestmates, who are on their way out into the saltpan. Such contacts potentially could convince the homing ant that it is near the nest and cause it to respond to a nest plume despite its remaining home vector. However, it was recently shown that contacts with nestmates do not affect an ant’s search behavior. Ants that, at the beginning of their search, were exposed to several nest mates displayed the same search pattern as control ants, which lacked this social input.^22^ Therefore, any effect of social contact on the willingness to follow the nest plume seems rather unlikely.

### Limitations of the study

Our study revealed that homing ants do not follow a nest plume when their PI tells them that they are far from home, while during the nest search, ants often follow the plume despite a long home vector. We were able to show that time outside the nest or exposure to heat stress does not affect the ants’ motivation to follow the plume. However, we tested only one specific vector length (i.e., 10 m distance from the nest). Future studies shall reveal at what vector length homing ants start to respond to the plume.

## Resource availability

### Lead contact

Requests for further information and resources should be directed to and will be fulfilled by the lead contact, Markus Knaden (mknaden@ice.mpg.de).

### Materials availability

This study did not generate new unique reagents.

### Data and code availability

• All raw data are available in the supplemental information (Data S1).
• This paper does not report original code.
• Additional information on the data reported in this article and its analysis is available from the lead contact upon request.

## Acknowledgments

We thank Lin Jiang for help in the field. The study was funded by the 10.13039/501100004189Max Planck Society.

## Author contributions

Conceptualization: R.S., J.E., and M.K.; data collection: R.S. and J.E.; data analysis: R.S., J.E., and M.K.; figures: R.S., J.E., and M.K.; first draft of the manuscript: R.S. and J.E.; editing of the manuscript, R. S., J.E., and M.K.: supervision: M.K.

## Declaration of interests

The authors declare no competing interests.

## Declaration of generative AI and AI-assisted technologies in the writing process

The authors declare that they have not used any AI-assisted technologies in the writing process.

## STAR★Methods

### Key resources table

**Table:** 

REAGENT or RESOURCE	SOURCE	IDENTIFIER
**Deposited data**		

All data provided in the manuscript and supplements	N/A	N/A

**Experimental models: Organisms/strains**		

Workers of the desert ant *Cataglyphis fortis*	N/A	Taxonomy ID: 606542

**Software and algorithms**		

Adobe Illustrator version 29.8.7 for figures	Adobe	www.adobe.com
GraphPad InStat version 3.1 for statistical analyses	GraphPad Prism Software	www.graphpad.com
LocusMaps version 5.25.1 for GPS tracking	Asamm Software	www.locusmap.app

**Other**		

Samsung Galaxy S22 for GPS tracking	Samsung Electronics	Model: SM-S901B

### Experimental model and study participant details

The study was performed with foragers of the desert ant *Cataglyphis fortis*. Like in all ants, foragers of this species are always female. As our experiments were performed in the field with freely moving ants, we are not aware of the age of the tested animals.

### Method details

#### Experimental site

Experiments were performed in August 2024 and June 2025 in a flat salt pan close to the Tunisian village Menzel Chaker (Latitude 34.957N, Longitude 10.410E). Experimental nests were situated in the center of the salt pan to avoid interference of neighboring nests at the feeder.

#### Homing ants follow their path-integration vector

In order to test for the interactions between path integration and nest-plume following, we removed obvious visible cues from the focus nest by flattening its nest hill and letting the ants adapt to this situation for 24 h. The nests were distantly isolated from other ant nests, to avoid testing foreign ants. On the next day the current wind direction was measured using a light plastic foil bound to the top of a stick. An artificial feeder with cookie crumbs was installed in 12 m distance from that nest with the nest-to-feeder direction being perpendicular to the wind direction. The wind direction in the salt pan during the day is rather stable, while there are mainly differences in wind directions between days. However, during the experimental days we always kept track of the wind direction, to make sure that the general wind direction had not changed. Foraging ants were then lured to this feeder by dispersing some cookie crumbs along the way resulting in many ants traveling back and forth between nest and feeder after about 1 h. In around 20 m distance from the nest a 12 m long line was drawn on the ground that ran parallel to the nest-to-feeder direction. Ants, which had collected a cookie crumb at the feeder, were captured and displaced at the start of the line. Due to their path-integration vector, they ran alongside the line and were then recaptured either after they had covered 2 m distance (i.e., when they still had a remaining path integration vector of 10 m length) or at the end of the 12 m line (i.e., when they had run off their path integration vector completely). The few ants that deviated more than one meter from the line were excluded as a conservative control measure, ensuring that only sufficiently trained individuals were tested, and to exclude any possibility that they originated from a distant foreign nest. The recaptured ants were then displaced 0.5 m downwind of their nest entrance and their behavior was observed. We drew a 1 m^2^ grid (mesh size,10 cm × 10 cm) around the nest entrance and video-recorded some of the ants’ runs for the later representation of example runs (Figure 2A_i-ii_). Here and elsewhere, the videos were later watched and the example paths were drawn by hand in Adobe Illustrator. We analyzed for all tested ants, whether they would follow the nest plume and finally would enter the nest entrance within 1 min (Figure 2B_i-ii_) although most of the ants that entered the nest arrived in less than 10 s (suppl. Data).

#### Searching ants ignore their path-integration vector

In a second set of experiments, we asked whether a search-derived PI vector inhibits the following of the nest plume also. Foraging ants from the focus nest were captured at the same feeder as before, were released at the remote 12 m line and were allowed to run off their PI vector completely (with again ants being discarded when they deviated from the line by more than 1 m). In about 20 m distance from this recapture point, we now drew a circle on the ground (radius, 10 m) and released individual ants that still kept their food crumb to the center of the circle. The ants started a systematic search for the nest immediately and the searching behavior of some ants was tracked using GPS (tracked with LocusMaps version 5.25.1 on a Samsung Galaxy S22) for the representation of example runs (Figure 1_iii-iv_). Under the local conditions (contact to 14–16 satellites) results in an accuracy of less than 2 m. However, this accuracy becomes even higher, if you do not consider the absolute position, but the relative positions during a single path that was recorded within a short time (see Figure 1A in Huber and Knaden^10^). At some point the ants extended their search and finally reached the edge of the 10 m circle, where they were captured again (i.e., these ants now had a search-derived PI vector of 10 m length). The ants were then displaced 0.5 m downwind either of their own nest (Figure 1_iii_) or of a foreign nest (Figure 1_iv_) and their behavior observed and analyzed as before (Figures 2A_iii-iv_ and 2B_iii-iv_). It was observed whether the ants entered their nest or continued their searching behavior since they had 10 m home vector left. The time until the ants entered the nest was measured. A search time of more than 60 s was defined as not entering the nest. The homecoming behavior was recorded, and the homecoming runs were drawn on paper, the 10 cm × 10 cm grid served as a reference. Ants with this treatment were not only placed next to their own nest entrance but also next to a foreign nest. The collected data was analyzed using a Fisher’s exact test with Bonferroni-Holm correction for repeated comparisons.

#### Time has no influence on foraging-derived PI

Our next question was whether the time the ants spent searching for their nest caused them to ignore their PI-vector and enter the nest. To test that, ants with cookie crumb were again captured at the feeder and were transferred to the remote 12 m line where they were allowed to run off 2 m of their PI-vector (Figure 3). They were then recaptured and placed into an arena for 10 min representing the searching time in the 10 m radius (Figure 3_iii_). From former experiments we concluded that such a short time would not result in heat stress for the ants. After 10 min the ants with cookie crumb were caught again and displaced back to the nest where they were released as before 0.5 m downwind of their own nest entrance. Their homing behavior was observed and video recorded for some example runs. Ants that did not take a food item after the time exposure were excluded from the analysis, to ensure that only ants with a homing motivation were tested.

#### Heat stress has no influence on foraging-derived PI

For exhibiting the limits of the foraging-derived PI and differentiating from the foraging-derived PI the influence of heat stress on the homing behavior was tested. Therefore, the ants with cookie crumb were again captured at the feeder and released at the 12 m line. They were allowed to run off 2 m of their PI-vector before they were captured again and set into an arena (Figure 3). The arenas were designed in a way that no shadow was covering the ground (using transparent plastic rings with 15 cm diameter) and the sand would be heated up by the desert-sun (Figure 3_iv-v_). In the arenas the ants were exposed to sun and heat for 60 min (Figure 3_iv_) or even 120 min (Figure 3_v_). After that time the ants were carefully recaptured and displaced 0.5 m downwind of their own nest entrance and their behavior was observed. We video-recorded some ants’ runs using a drawn grid (mesh size, 10 cm × 10 cm) around the nest entrance for later representation of some example runs. Finally, we analyzed whether the tested ants would follow their foraging-derived PI vector or, after extensive heat stress, follow the nest plume and enter the nest entrance within 1 min. Ants that did not take a food item after the time exposure were excluded from the analysis, to ensure that only ants with a homing motivation were tested.

### Quantification and statistical analysis

We always compared the plume following behavior of the ants of the six different treatments (Figures 1B_ii-iv_ and 2B_i-iii_) with those of the control ants (Figure 2B_i_) that had a foraging-derived PI vector of 10 m length. We used a Fisher’s exact test and adjusted the level of significance using a Bonferroni-Holm correction for repeated comparisons.

## Supplemental information

Document S1. Table S1

Data S1. Contains an Excel file with all raw data

## References

[1] Packmor F., Kishkinev D., Zechmeister T., Mouritsen H., Holland R.A. (2024). Migratory birds can extract positional information from magnetic inclination and magnetic declination alone. Proc. Biol. Sci..

[2] Mouritsen H., Feenders G., Liedvogel M., Kropp W. (2004). Migratory birds use head scans to detect the direction of the earth's magnetic field. Curr. Biol..

[3] Dreyer D., Frost B., Mouritsen H., Günther A., Green K., Whitehouse M., Johnsen S., Heinze S., Warrant E. (2018). The earth’s magnetic field and visual landmarks steer migratory flight behavior in the nocturnal Australian bogong moth. Curr. Biol..

[4] Guerra P.A., Gegear R.J., Reppert S.M. (2014). A magnetic compass aids monarch butterfly migration. Nat. Commun..

[5] Guilford T., Taylor G.K. (2014). The sun compass revisited. Anim. Behav..

[6] Czaczkes T.J., Weichselgartner T., Bernadou A., Heinze J. (2016). The effect of trail pheromone and path confinement on learning of complex routes in the ant *Lasius niger*. PLoS One.

[7] Butterfield T., Bacon J., Hill E.M. (2025). Identification of trail following and alarm pheromones of *Lasius Flavus* using bioassay-directed fractionation. J. Chem. Ecol..

[8] van Oudenhove L., Billoir E., Boulay R., Bernstein C., Cerdá X. (2011). Temperature limits trail following behaviour through pheromone decay in ants. Naturwissenschaften.

[9] Müller M., Wehner R. (1988). Path integration in desert ants, *Cataglyphis fortis*. Proc. Natl. Acad. Sci. USA.

[10] Huber R., Knaden M. (2015). Egocentric and geocentric navigation during extremely long foraging paths of desert ants. J. Comp. Physiol. A Neuroethol. Sens. Neural Behav. Physiol..

[11] Wittlinger M., Wehner R., Wolf H. (2006). The Ant Odometer: Stepping on Stilts and Stumps. Science.

[12] Buehlmann C., Hansson B.S., Knaden M. (2012). Path Integration Controls Nest-Plume Following in Desert Ants. Curr. Biol..

[13] Knaden M., Wehner R. (2004). Path integration in desert ants controls aggressiveness. Science.

[14] Müller M., Wehner R. (1994). The hidden spiral: systematic search and path integration in desert ants, Cataglyphis fortis. J. Comp. Physiol..

[15] Lima S.L., Dill L.M. (1990). Behavioral decisions made under the risk of predation: a review and prospectus. Can. J. Zool..

[16] Gehring W.J., Wehner R. (1995). Heat shock protein synthesis and thermotolerance in *Cataglyphis*, an ant from the Sahara desert. Proc. Natl. Acad. Sci. USA.

[17] Zaguri M., Zohar Y., Hawlena D. (2018). Considerations used by desert isopods to assess scorpion predation risk. Am. Nat..

[18] Zaguri M., Hawlena D. (2020). Odours of non-predatory species help prey moderate their risk assessment. Funct. Ecol..

[19] Walls M.L., Layne J.E. (2009). Direct evidence for distance measurement via flexible stride integration in the fiddler crab. Curr. Biol..

[20] Hemmi J.M., Zeil J. (2003). Burrow surveillance in fiddler crabs. I. Description of behaviour. J. Exp. Biol..

[21] Deeti S., Cheng K. (2024). Desert ant (Melophorus bagoti) dumpers learn from experience to improve waste disposal and show spatial fidelity. Insects.

[22] Bollig A., Freire M., Bücking K., Kühnapfel J., Knaden M. (2026). Aggressive conflict as a transient and distributed landmark in homing desert ants. bioRxiv.

