# Supplementary material for: Context-dependent influence of path integration on nest plume following in desert ants
Source: iScience. 2026 Jun 25;29(7):116523. doi: 10.1016/j.isci.2026.116523 (PMC13377868; doi:10.1016/j.isci.2026.116523)
Supplement: Document S1. Table S1 [file mmc1.pdf]

iScience, Volume *29*

Supplemental information

Context-dependent influence of path integration on nest plume following in desert ants

Richard Spehr, Johanna Eschenhagen, and Markus Knaden

Control	Treatment	p-value Fisher's exact test	corrected level of significance (alpha)	Odds ratio	95% confidence intervall
Fig. **2Bi** tested vs	**2Bii**	<0.0001	0,0100	0,0022	0.0003 to 0.0196
	**2Biii**	<0.0001	0,0083	0,0329	0.0102 to 0.1066
	**2Biv**	0,0125	0,0125	0,1515	0.0371 to 0.6188
	**3Bi**	0,1202	0,05	0,3030	0.0796 to 1.1540
	**3Bii**	0,1202	0,025	0,3030	0.0796 to 1.1540
	**3Biii**	0,0322	0,0167	0,2399	0.0659 to 0.8734

Table S1. Statistical comparisons of data shown in Fig. 2 and Fig. 3 of the manuscript. All treatments (**2B**$_{ii}$, homing ants, Pi vector: 0 meter; **2B**$_{iii}$, searching ants, Pi vector: 10 meter; **2B**$_{iv}$, searching ants, Pi vector: 10 meter, tested at foreign nest; **3B**$_{i}$, homing ants, Pi vector: 10 meter, tested after 10 min sun exposure; **3B**$_{ii}$, homing ants, Pi vector: 10 meter, tested after 60 min sun exposure; and **3B**$_{iii}$, homing ants, Pi vector: 10 meter, tested after 120 min sun exposure) were tested against data obtained from control group (**2B**$_{i}$, homing ants, Pi vector 10 meter). Data were tested with Fisher’s exact test with Bonferroni-Holm correction for repeated comparisons of the level of significance (α). Data with significant difference (where $p <$ corrected α) are highlighted in grey.
